# Exploring the gut microbiome and immunological landscape in kidney cancer: a Mendelian randomization analysis

**DOI:** 10.3389/fimmu.2024.1459967

**Published:** 2024-08-29

**Authors:** Shihui Lv, Qian Guo, Yuhan He, Zhixian Yu, Xianjing Zhan, Hang Li, Yue Pan

**Affiliations:** ^1^ Department of Urology, First Affiliated Hospital of Wenzhou Medical University, Wenzhou, China; ^2^ Department of Rhinology, FirstAffiliated Hospital of Zhengzhou University, Zhengzhou, China; ^3^ Department of Urology, YongKang First People’s Hospital of Hangzhou Medical College, Yongkang, China

**Keywords:** kidney cancer, immune system, gut microbiota, Mendelian randomization, inflammatory proteins

## Abstract

**Introduction:**

Kidney cancer (KC) is a significant health burden globally, with over 400,000 new cases estimated in 2020. The prognosis of KC is influenced by various factors, including tumor spread, pathological characteristics, and molecular genetic changes. Recent studies have emphasized the involvement of gut microbiota and the immune system’s contribution in the onset of KC. This extensive research endeavor sought to investigate the potential associations between diverse immune cell phenotypes, specific gut microbiota species, and their impact on the risk of developing KC, alongside the examination of circulating inflammatory proteins.

**Methods:**

Adhering to the STROBE-MR guidelines, our investigation involved a two-stage Mendelian randomization (2SMR) analysis grounded on three fundamental assumptions: relevance, independence, and exclusion restriction. The exposure data utilized in this study originated from genome-wide association studies (GWAS) specifically designed to explore immune traits, inflammatory proteins, and gut microbiota compositions.

**Results:**

Our analysis identified 25 immune phenotypes, 4 circulating inflammatory proteins, and 12 gut microbiota features that exhibited significant causal associations with KC (P < 0.05). 10 immune phenotypes were protective against KC, while 15 were risk factors. Among the inflammatory proteins, CCL28 and IL-2 were protective, whereas FGF-23 and β-NGF were risk factors. Gut microbiota features associated with reduced KC risk included biosynthetic pathways involving amino acids and specific bacterial genera, whereas others, like Butyrivibrio crossotus and Odoribacter splanchnicus, were risk factors.

**Conclusion:**

Immune, inflammatory, and gut microbiota factors impact KC development. Identified factors hint at biomarkers and therapeutic targets. It is very important to understand the relationship between these factors and KC.

## Introduction

In 2020, globally, it was estimated that over 400,000 individuals were diagnosed with kidney cancer (KC) (1). Due to gender differences in incidence, KC ranks in the top ten most common cancers among men and in the top fifteen among women. The prognosis of KC patients is determined by numerous factors, encompassing the tumor’s local and distant spread, its pathological features, the genetic landscape at the molecular level, and the clinical signs and symptoms presented by the patient. In cases of localized KC, conventional risk assessment is typically based on the disease’s anatomical spread, with various sophisticated scoring systems established to forecast patient outcomes following curative local therapies (2).

In 2022, Chinese researchers conducted in-depth studies on clear cell renal cell carcinoma (ccRCC) using multi-omics sequencing technology. They found that VHL and PBRM1 genes have the highest mutation rates, revealing the metabolic and immune heterogeneity of ccRCC and classifying it into three subtypes: GP1, GP2, and GP3. Among them, the GP1 subtype is highly invasive and metastatic, with poor prognosis (3). The study also discovered the role of nicotinamide N-methyltransferase in DNA repair, which accelerates the development of ccRCC. Additionally, the study shed more light on the heterogeneity within tumors and the pivotal role of CD8^+^ T cells within the tumor microenvironment, thereby revealing novel opportunities for therapeutic intervention (4), this suggests that the influence of immune cells on KC should not be ignored. Gene expression analysis also defined pathological molecular subtypes related to prognosis, promoting the development of personalized treatment for renal cell carcinoma (RCC).

The role of immune cells in the tumor microenvironment has been extensively studied, and they are involved in tumor immune surveillance and immune escape by modulating the immune response. It is of great significance for the occurrence and development of KZ to study it deeply. In addition, inflammatory factors secreted by immune cells, such as cytokines and chemokines, are also Bridges between inflammation and tumorigenicity. They are not only involved in the regulation of inflammatory response, but also affect the proliferation, invasion and metastasis of tumor cells. The abnormal expression of inflammatory factors is closely related to the occurrence and development of many cancers, including kidney cancer.

Changes in lifestyle, as well as the increasing prevalence of systemic diseases such as obesity and hypertension, are associated with the risk of KC development. Cigarette smoking (5, 6), as well as being overweight and obese (7, 8), along with hypertension (9–11), are recognized as significant but moderate contributors to the risk of developing KC. In contrast, the intake of alcohol in mild to moderate amounts appears to exert a protective influence, potentially slowing the progression of KC (12, 13). In the past decade, research on the pathogenesis of KC has made some significant progress. The gut microbiota may play a role in the development of tumors by influencing the metabolic, immune and inflammatory responses of the host. The relevance of specific gut microbiome communities to tumor risk and treatment response is becoming a focus of research. In addition, a growing body of research suggests that lifestyle changes may have a significant impact on the gut microbiota. Therefore, gut microbiota may also be an important cause of the occurrence and development of KC, and it is of great clinical significance to further explore its internal correlation.

The aim of this study was to explore the potential causal relationship between immune cells, inflammatory factors, and gut microbiota and KC using Mendelian randomization. By analyzing the association between genetic variants of these biomarkers and KC risk, we hope to shed light on their possible mechanisms of action in kidney cancer development and provide new insights into future prevention and treatment strategies.

## Materials and methods

### Research methodology

Our investigation meticulously assessed the causal links between 731 distinct immune cell phenotypes, 91 types of circulating inflammatory proteins, and 412 species of gut microbiota in relation to the risk of KC, adhering to the STROBE-MR guidelines (14). This comprehensive approach utilized a two-step Mendelian randomization (2SMR) analysis framework, grounded in three fundamental assumptions that guaranteed the study’s methodological rigor and the reliability of its findings.

In our research, we strictly followed the core tenets of MR analysis:

(1) Relevance Tenet: We ensured that the chosen genetic variants, especially single-nucleotide polymorphisms (SNPs), used as instrumental variables, had a substantial and consistent link to the exposure factors we were studying, including immune phenotypes, inflammatory proteins, and gut microbiota. This linkage is essential for establishing a theoretical basis to evaluate how these exposures might affect the outcome of interest, which in this case is the risk of KC. (2) Independence Tenet: We required that these genetic variants be free from the influence of confounding factors, akin to their random distribution in natural reproduction, thus ensuring that our analysis results were unbiased and reliable. (3) Exclusion Restriction Tenet: We emphasized that the impact of genetic variants on the outcome should be exclusively through the exposures of interest, ruling out any alternative pathways. This focus reinforced the causal interpretation of the relationship between the exposures and the outcome.

**Figure 1:**
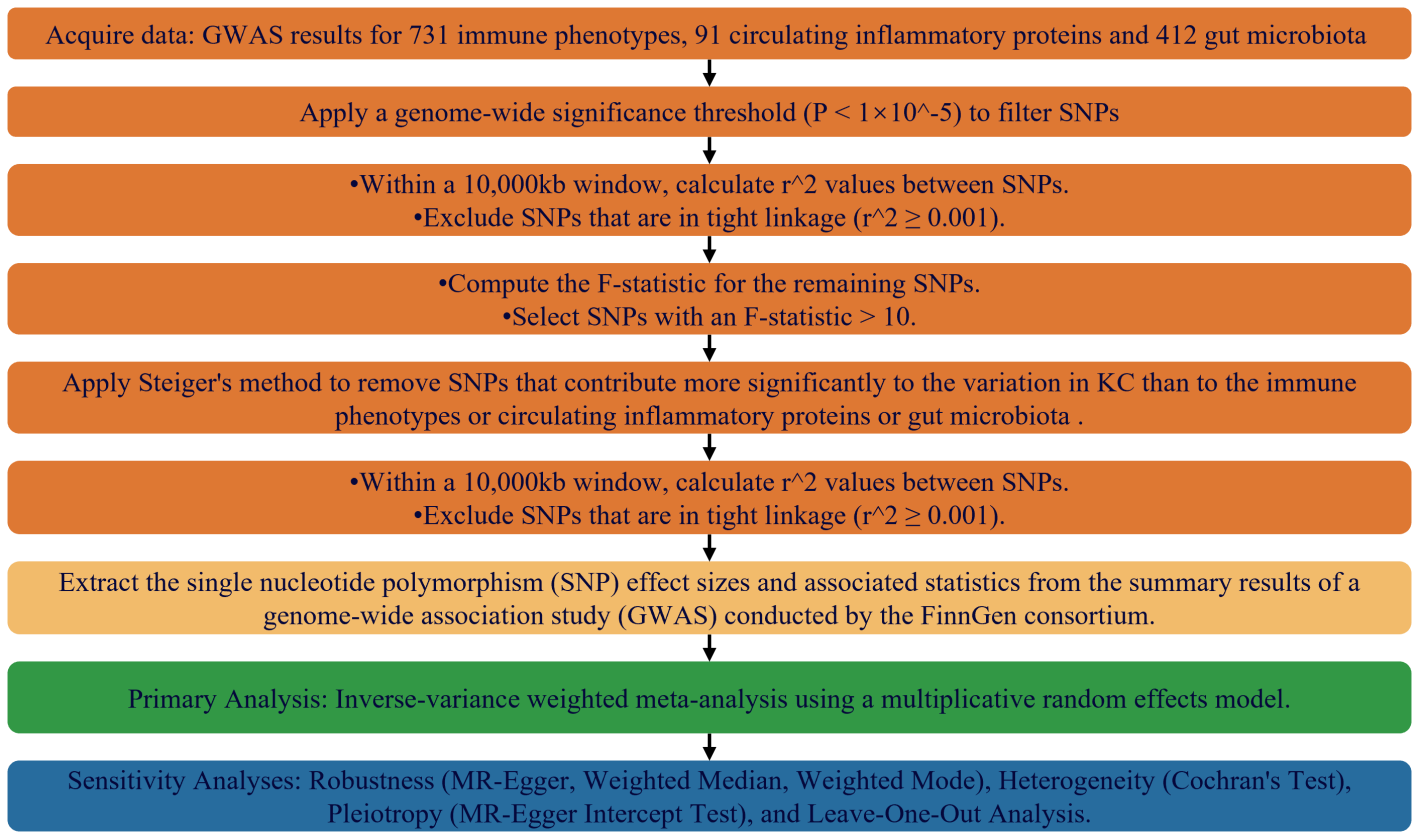
Flowchart Depicting the MR Framework for Evaluating Causal Relationships Among Immunophenotypes, Inflammatory Markers, Gut Microbiome Features, and KC.

In the process of selecting instrumental variables, we applied three stringent criteria: a clear link to exposure factors, independence from confounding influences, and exclusive mediation of the outcome through these factors. To encapsulate, the study’s design, execution, and careful selection of genetic instrumental variables, as detailed in **Figure 1**, were carried out with transparency, scientific discipline, and methodological soundness.

### Data sources for exposure GWAS

Our research is anchored in a wide-ranging genome-wide association study (GWAS) that provided immunological data, documented with accession numbers from GCST90001456 to GCST90002025 (https://www.ebi.ac.uk/gwas), and covering 731 unique immunophenotypes (15). The scope of the immune cell types examined is vast, including but not limited to B cells, dendritic cells, T cells in various developmental stages, monocytes, myeloid cells, and both TBNK (a combination of T cells, B cells, and natural killer cells) and Treg cells, with a special focus on the morphological aspects of dendritic and TBNK cells. The initial GWAS analysis was conducted on 2,218 individuals of European ancestry, meticulously designed to prevent any cohort overlap. High-density arrays were employed to genotype around 22 million single-nucleotide polymorphisms (SNPs), with imputation based on a reference panel derived from Sardinian sequences. Additionally, the study incorporated data on 91 circulating inflammatory proteins from a separate GWAS comprising 14,824 participants of European descent, aimed at pinpointing genetic variants that affect cytokine levels in the bloodstream (16). To round out our genetic analysis, variant data pertaining to the gut microbiota were sourced from the MiBioGen consortium (https://mibiogen.gcc.rug.nl/menu/main/home/) (17).

### Selection of genetic instrumental variables

For the identification of genetic IVs associated with immune traits, our study implemented a rigorous significance threshold set at 1×10^-5^. This stringent criterion extended to the linkage disequilibrium (LD) pruning process for single-nucleotide polymorphisms (SNPs), where SNPs were excluded if they had an LD r^2^ value greater than 0.001 within a 10,000 kb window. The IVs designated for circulating inflammatory proteins adhered to the same threshold and underwent the same LD pruning protocol.

To counteract the influence of weak IVs, we evaluated the F-statistic for every genetic variant considered as an IV. SNPs with an F-statistic below the threshold of 10 were deemed weak and were consequently omitted from our analysis, a measure taken to safeguard the robustness of our results. Furthermore, in consideration of the exclusion scenario for kidney malignant neoplasms, we applied a relaxed significance threshold of 5×10^-5^ for the selection of IVs, allowing for a broader selection in this specific context.

### Genetic associations for outcomes

In pursuit of examining the genetic connections to KC, we have gathered extensive data from the renowned FinnGen consortium (https://www.finngen.fi/en/access_results), accessible via their online platform. Our dataset was sourced from the consortium’s tenth release of GWAS summaries, made publicly accessible on the 18th of December, 2023. Utilizing this robust collection of data, we have conducted an exhaustive investigation into the genetic factors associated with KC.

### Statistical analysis strategy

In this research, we applied a stringent two-sample Mendelian randomization (2SMR) analysis to meticulously dissect the complex interrelationships among diverse immune cell phenotypes, levels of circulating inflammatory proteins, features of the gut microbiota, and the risk of KC onset. We prioritized the inverse variance-weighted (IVW) methodology, which is widely recognized and applied in MR research. To bolster the robustness of our discoveries, we additionally employed MREgger regression, the weighted median technique, and the weighted mode method as complementary validation strategies in conjunction with our primary analytical approach. Additionally, we conducted a detailed assessment of potential vertical pleiotropy (i.e., reverse causality in gene-disease associations) through the intercept analysis of MR-Egger regression. The statistical analysis and data visualization processes in this study were performed within the Rstudio environment, using version 4.3.1. To facilitate the execution of the MR analysis, we capitalized on the robust functionality of the “Two Sample MR” and “Mendelian Randomization” R packages. These packages proficiently facilitated the integration of datasets and the implementation of MR analytical methods, thereby enhancing the precision and efficiency of our study.

Furthermore, we comprehensively evaluated the heterogeneity of our results using Cochran’s Q statistical test, aiming to uphold scientific rigor and maintain objectivity. In addition, p less than 0.05 was deemed as significant.

## Result

### The identification of IVs

After a series of rigorous screening criteria, this study identified a total of 10,724 single nucleotide polymorphisms (SNPs) and designated them as instrumental variables (IVs) for exposure factors such as immune cell characteristics, inflammatory proteins, and gut microbiota features. To ensure the validity of these IVs, we evaluated them using the F-statistic, and the results showed that all selected IVs had F-values exceeding 10, indicating that they are strong instrumental variables, thus reducing the risk of estimation bias due to weak instrumental variables. These detailed evaluation results have been presented in **Supplementary Tables S1–S3** in the supplementary materials.

### Overview

In our study, through a detailed analysis of 2,218 immune cells, we identified their causal roles in the development of KC (possibly referring to a certain disease or condition). Employing a P-value threshold of less than 0.05 as our screening criterion, we identified 10 immune phenotypes that demonstrate an association with an elevated risk of KC. These include the percentage of Activated & secreting CD4 regulatory T cells among CD4 regulatory T cells, CD80 expression on granulocytes, CD86 expression on monocytes, CD3 expression on CD28^+^ CD45RA^-^ CD8^+^ T cells, the absolute count of CD62L^-^ plasmacytoid Dendritic Cells, CD45 expression on CD33^+^ HLA DR^+^ CD14^-^ cells, CD4 expression on HLA DR^+^ CD4^+^ T cells, the absolute count of CD4-CD8- T cells, the percentage of CD28^+^ CD4^-^CD8^-^ T cells among T cells, and the absolute count of CD127^-^ CD8^+^ T cells. Our investigation further pinpointed ten immune phenotypes that may exhibit a protective effect against the development of KC. The protective immune phenotypes encompassed the following: FSC-A expression on CD14^+^ monocytes, CD27 expression on CD20^-^ B cells, the percentage of CD86^+^ plasmacytoid Dendritic Cells among Dendritic Cells, CD20 expression on IgD^+^ CD38^-^ unswitched memory B cells, CD20 expression on IgD^+^ CD38^-^ naive B cells, the absolute count of CD11c^+^ HLA DR^++^ monocytes, CD62L expression on CD62L^+^ myeloid Dendritic Cells, BAFF-R expression on CD20^-^ CD38^-^ B cells, CD4 expression on naive CD4^+^ T cells, as well as various expressions and percentages related to T cells and Natural Killer T cells, including HVEM on T cells, CD4 on CD39^+^ resting CD4 regulatory T cells, CD45 on Natural Killer T, and resting CD4 regulatory T cell percentages among CD4^+^ T cells and CD4 regulatory T cells, respectively, in addition to HLA DR on CD14^+^ CD16^+^ monocytes.

Our study delved into the functional roles of four plasma inflammatory molecules in the context of KC. Notably, we discovered that heightened concentrations of C-C motif chemokine 28 and Interleukin-2 exhibited protective effects against the onset of KC. Conversely, we identified elevated levels of Fibroblast growth factor 23 and beta-nerve growth factor as significant risk factors for KC, with statistical significance confirmed at a p-value threshold of less than 0.05.

Furthermore, our investigation unveiled that 12 distinct characteristics of gut microbiota contribute to the etiology of KC. Our research specifically identified seven gut microbiota features that serve as protective factors against KC: the superpathways for L-lysine, L-threonine, and L-methionine biosynthesis II (PWY.724), sucrose degradation III via sucrose invertase (PWY.621), dTDP-L-rhamnose biosynthesis I (DTDPRHAMSYN.PWY), a specific strain of Desulfovibrio piger belonging to the Desulfovibrionaceae family (k_Bacteria.p_Proteobacteria.c_Deltaproteobacteria.o_Desulfovibrionales.f_Desulfovibrionaceae.g_Desulfovibrio.s_Desulfovibrio_piger), the glyoxylate cycle (PWY_GLYOXYLATE.BYPASS), and the superpathway for menaquinol-6 biosynthesis I (PWY.5850). Conversely, six gut microbiota features were identified as risk factors for KC development: Butyrivibrio crossotus, a member of the Lachnospiraceae family (k_Bacteria.p_Firmicutes.c_Clostridia.o_Clostridiales.f_Lachnospiraceae.g_Butyrivibrio.s_Butyrivibrio_crossotus), Odoribacter splanchnicus, belonging to the Porphyromonadaceae family (k_Bacteria.p_Bacteroidetes.c_Bacteroidia.o_Bacteroidales.f_Porphyromonadaceae.g_Odoribacter.s_Odoribacter_splanchnicus), Sutterella wadsworthensis, a member of the Sutterellaceae family (k_Bacteria.p_Proteobacteria.c_Betaproteobacteria.o_Burkholderiales.f_Sutterellaceae.g_Sutterella.s_Sutterella_wadsworthensis), the genus Odoribacter (k_Bacteria.p_Bacteroidetes.c_Bacteroidia.o_Bacteroidales.f_Porphyromonadaceae.g_Odoribacter), the stearate biosynthesis II pathway in bacteria and plants (PWY.5989), and Alistipes shahii, a member of the Rikenellaceae family (k_Bacteria.p_Bacteroidetes.c_Bacteroidia.o_Bacteroidales.f_Rikenellaceae.g_Alistipes.s_Alistipes_shahii).

According to the Steiger test, all p-values for the 25 distinct immune cell types, 4 circulating proinflammatory molecules, and 12 gut microbiota characteristics, which we have determined to hold a causal link with KC, fall below the significance threshold of 0.05. This outcome signifies that our findings do not manifest reverse causality, indicating the authenticity of the associations we have uncovered. A concise summary of these results is provided in **Supplementary Tables S4–S6**.

### The causal association between the attributes of immune cells and the development or progression of KC

**Figure 2:**
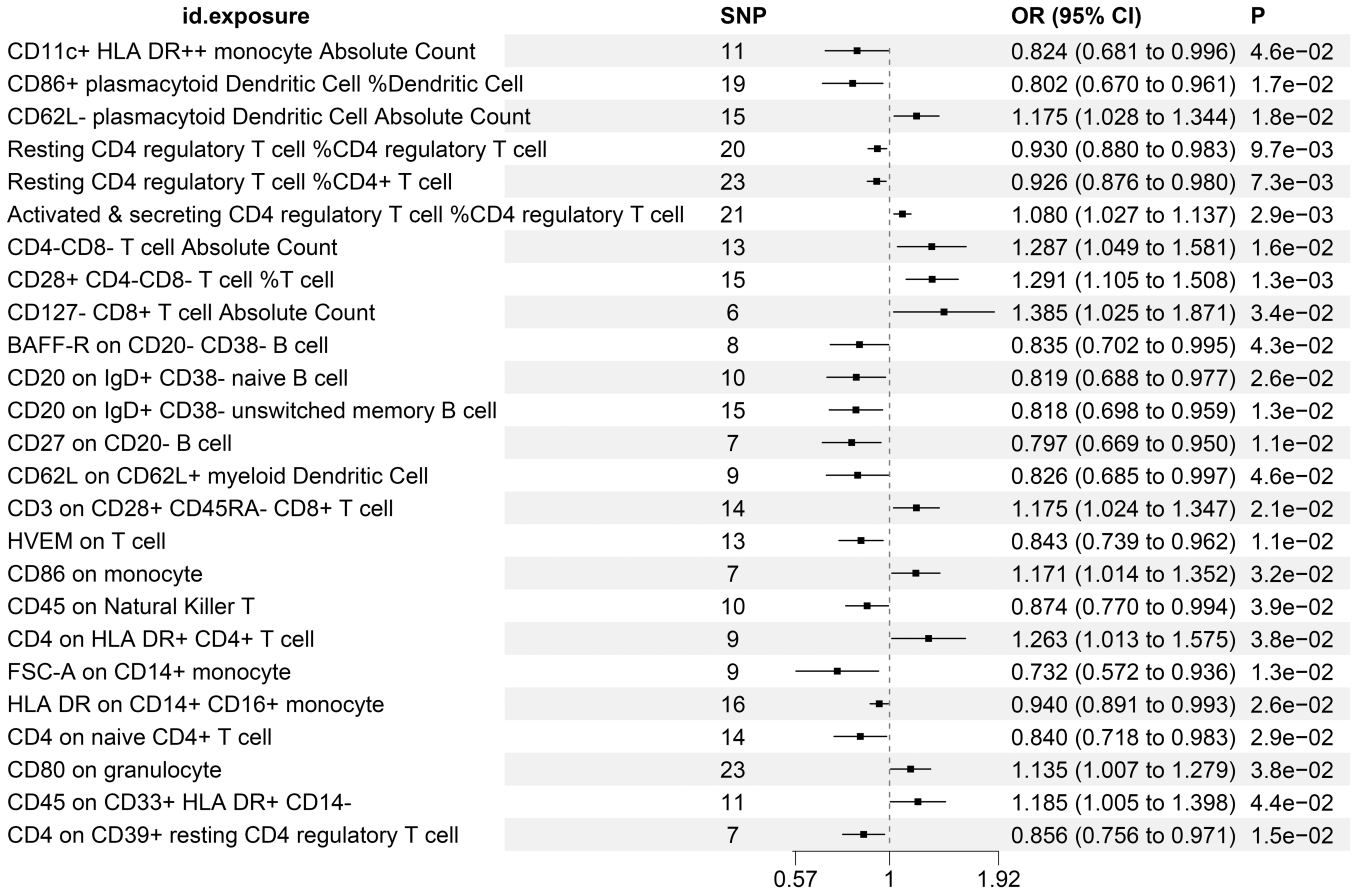
MR Examination Uncovers Causal Relationships Between Immune Cell Phenotypes and KC, Highlighting IVW Outcomes with Odds Ratios, Confidence Intervals, and Associated SNPs.

**Supplementary Table S7** enumerates 25 immune cell phenotypes that have been meticulously selected utilizing the IVW methodology. The causal linkages between these identified phenotypes and KC have been rigorously examined and have successfully surpassed a statistical significance threshold, as evidenced by P-values less than 0.05. **Figure 2** provides a comprehensive consolidation of the outcomes stemming from the MR analysis, which assesses the relationship between these immune phenotypes and KC. Specifically, the figure employs a forest plot to visually represent and summarize these findings.

**Figure 3:**
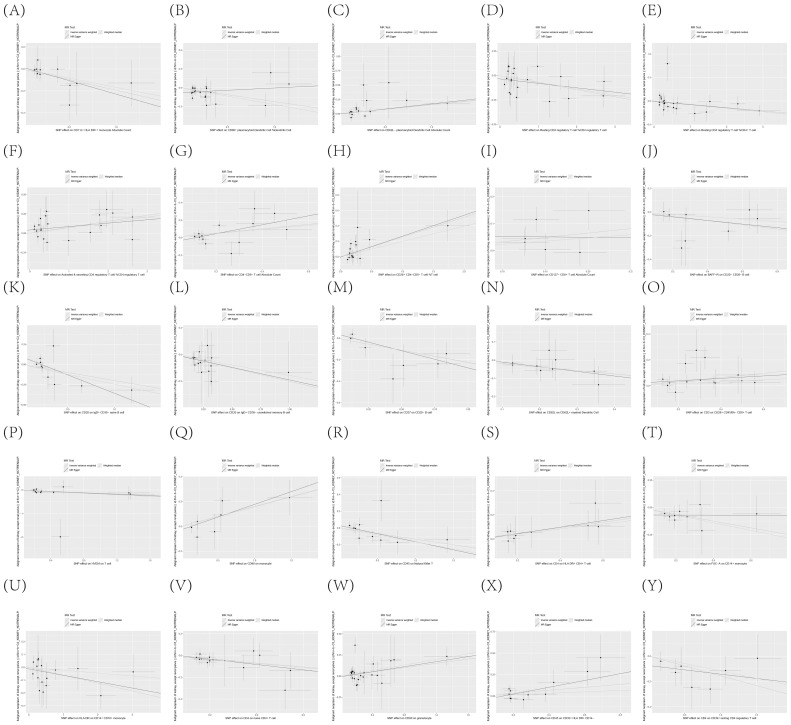
**(A–Y)** Dispersion Diagram Illustrating the Causal Connection Between Immune Cell Phenotypes and the Occurrence of KC, Labeled with Pertinent SNPs.

**Figure 4:**
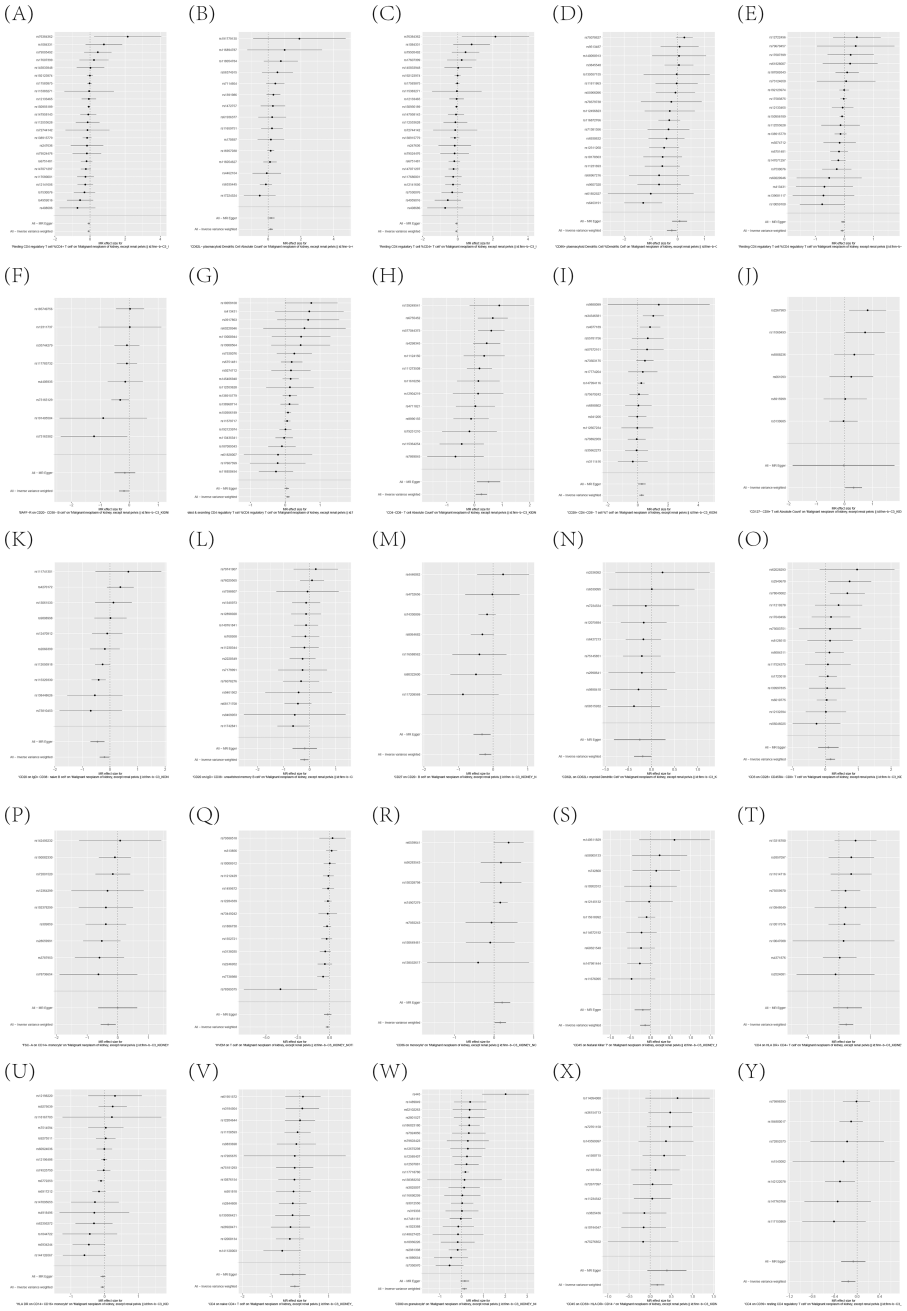
**(A–Y)** Leave-one-out analysis substantiates the strength and reliability of the causal link between immune cell phenotypes and KC.

To further corroborate the robustness of our research findings, we have presented them in a scatter plot within **Figure 3**, offering an additional layer of validation. Moreover, to mitigate potential biases and uphold the reliability of our results, we conducted three distinct types of sensitivity analyses: a leave-one-out analysis, Cochran’s Q test to assess heterogeneity, and the MR-Egger regression method for evaluating horizontal pleiotropy. The outcomes of these analyses are systematically presented in **Figure 4**, showcasing the leave-one-out analysis plot; **Supplementary Table S8**, detailing the results of Cochran’s Q test for heterogeneity; and **Supplementary Table S9**, reporting the analysis outcomes pertaining to horizontal pleiotropy. Collectively, these analyses provide compelling evidence that reinforces the robustness and credibility of our research findings.

### Significant connections discovered between circulating inflammatory proteins and the risk of KC

**Figure 5:**
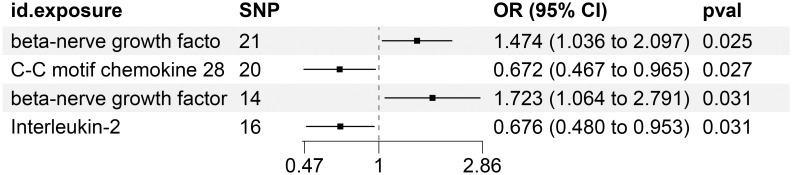
MR Analysis Investigates the Causality Between Circulating Inflammatory Proteins and KC Utilizing IVW Outcomes with Odds Ratios, Confidence Intervals, and Associated SNPs.

**Figure 6:**
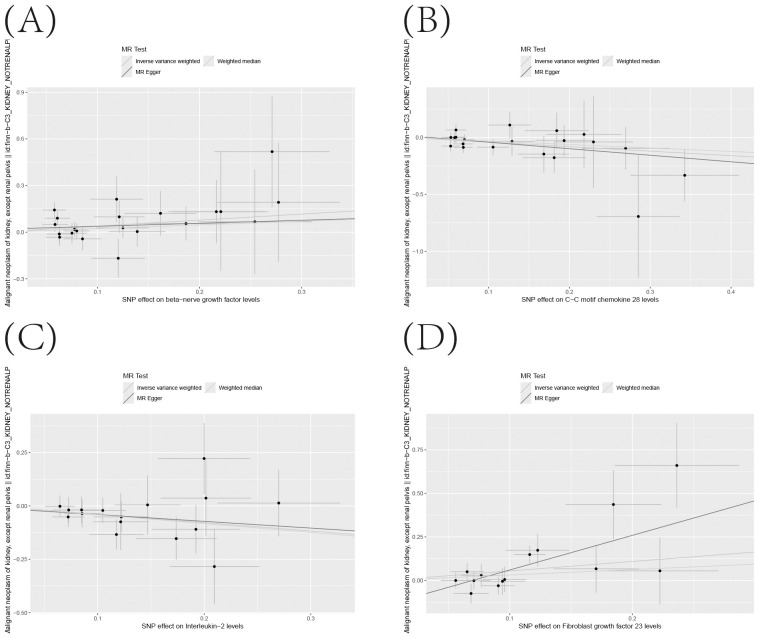
**(A–D)** Scatter Plot Demonstrating the Causality Between Circulating Levels of Inflammatory Proteins and the Incidence of KC, with Annotations for Significant SNPs.

**Supplementary Table S10** precisely outlines the statistically significant relationships (at P < 0.05 using the IVW method) between 4 systemic inflammatory biomarkers and KC. **Figure 5**, formatted as a blobbogram, concisely encapsulates the results of the MR analysis concerning the association between these 4 systemic inflammatory biomarkers and KC. The strength and consistency of these findings are further emphasized by their presentation in **Figure 6**.

**Figure 7:**
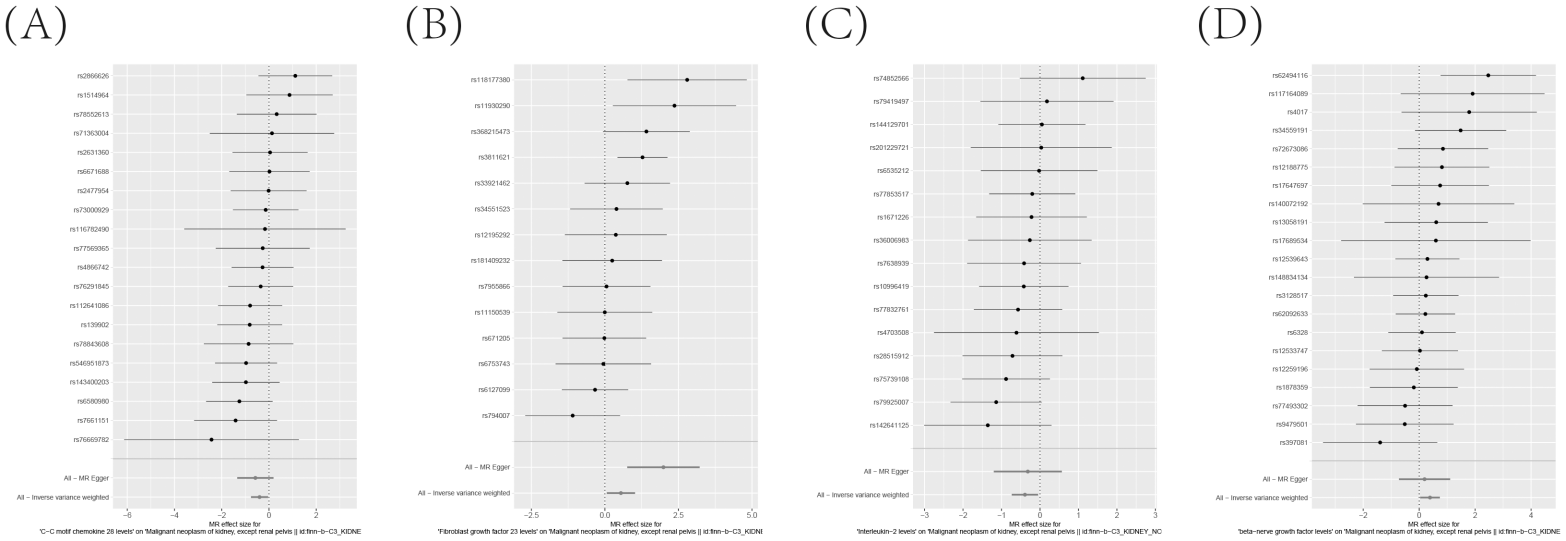
**(A–D)** Leave-one-out analysis Validates the Consistency and Strength of the Causal Connection Between Inflammatory Markers in the Bloodstream and the Development of KC.

To ensure the robustness and reliability of our findings beyond the primary analysis, we have conducted a comprehensive array of sensitivity analyses. These include a leave-one-out method, Cochran’s Q test to assess heterogeneity, and the MR-Egger approach for detecting horizontal pleiotropy. The outcomes of these rigorous evaluations are meticulously presented in **Figure 7**, showcasing the leave-one-out analysis, and in **Supplementary Tables S11, S12**, detailing the results of Cochran’s Q test and horizontal pleiotropy analysis, respectively. By incorporating these analyses, we have further strengthened the validity and credibility of our discoveries.

**Figure 8:**
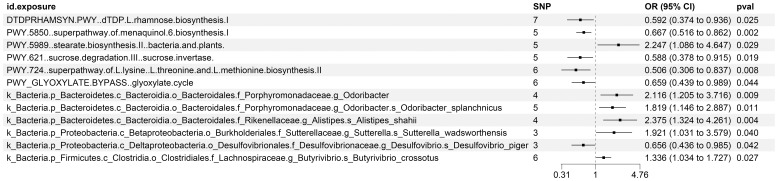
MR Study Exposes the Causality Between Intestinal Microbes and KC Using IVW Outcomes with Odds Ratios, Confidence Intervals, and Associated SNPs.

**Figure 9:**
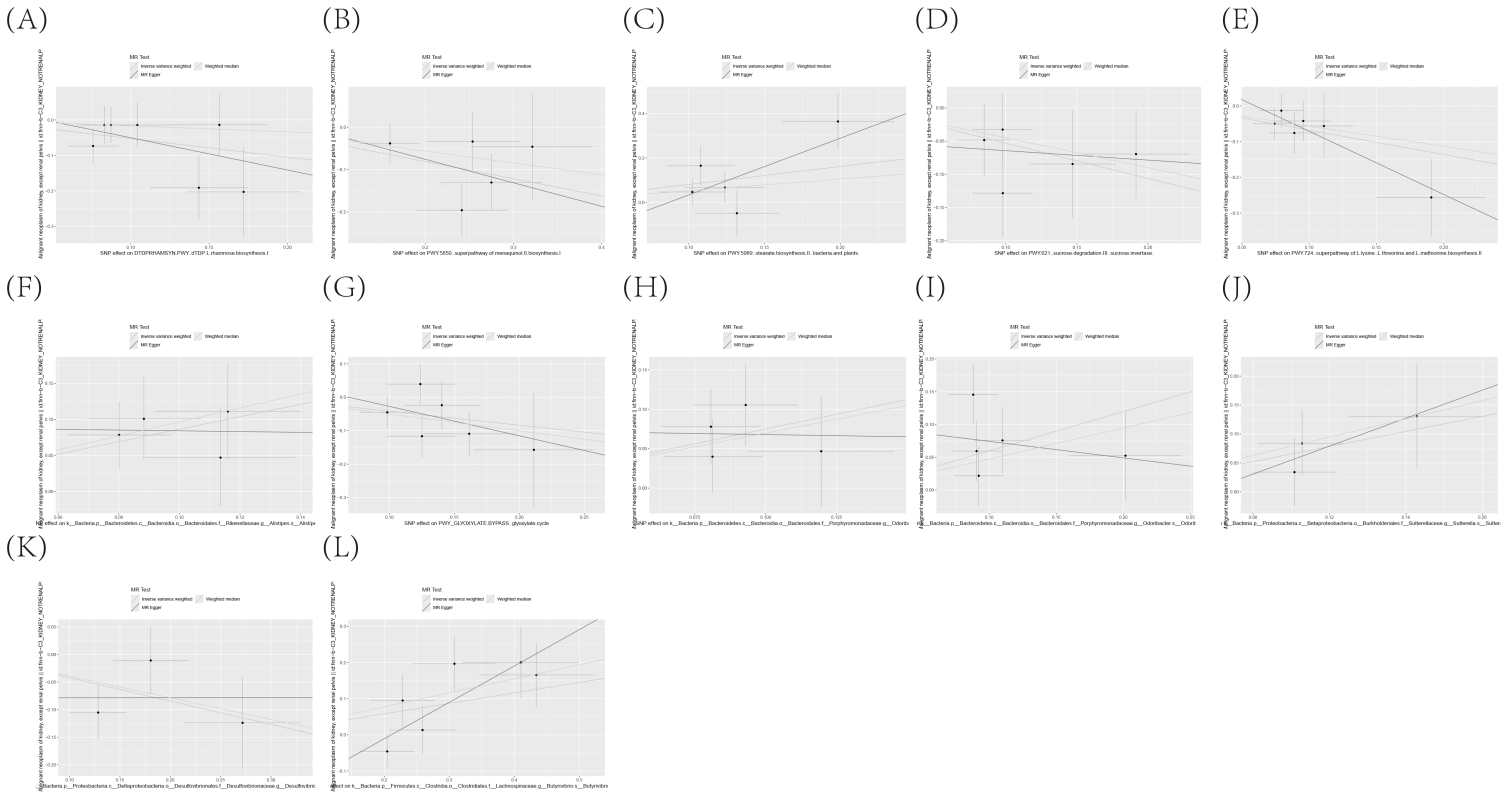
**(A–L)** Scatter plot delineating the causal relationship between gut microbiota composition and the incidence of KC, with annotations indicating the pertinent SNPs.

**Figure 10:**
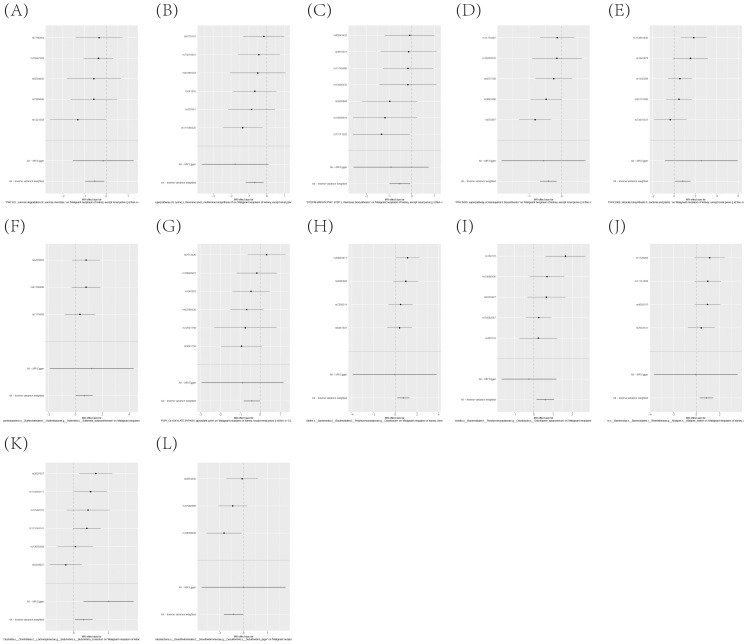
**(A–L)** Leave-one-out analysis affirms the robust causal relationship between gut microbiota and the onset of KC.

The existence of causal connections between the attributes of gut microbiota and KC was also analyzed. **Supplementary Table S13** presents a comprehensive exploration of the potential causal relationships between twelve pivotal characteristics of gut microbiota and the clinical manifestations of KC, as uncovered through a MR framework that employs the IVW method. This analysis identifies statistically significant associations (P < 0.05) between the selected gut microbiota features and KC outcomes. **Figure 8** visually summarizes the estimated effects and their confidence intervals of the associations between these gut microbial features and KC, offering an overview of their complex interactions. To further consolidate the robustness of these findings, we introduce **Figure 9** as supplementary evidence, which visually reinforce the stability and consistency of the strength of associations between gut microbiota characteristics and KC. Furthermore, to comprehensively evaluate the robustness of our findings, we applied a suite of rigorous sensitivity analysis techniques. Among these, we conducted leave-one-out analyses to evaluate the influence of individual studies on the overall conclusions, with the results of this analysis illustrated in **Figure 10**. Additionally, we employed Cochran’s Q test to scrutinize for heterogeneity across studies, with a detailed account of these findings presented in **Supplementary Material S14**. Furthermore, the MR-Egger method was utilized to detect and adjust for potential biases arising from horizontal pleiotropy, and the assessment of horizontal pleiotropy is thoroughly documented in **Supplementary Material S15**. This in-depth analysis not only enriches our understanding of the gut microbiota-KC association mechanisms but also significantly enhances the robustness and credibility of the conclusions drawn from this study.

## Discussion

Our study integrated extensive individual data and multiple aggregated GWAS results, revealing significant causal associations (all with P-values less than 0.05) between 25 immune phenotypes, 4 circulating inflammatory proteins, and 12 gut microbiota characteristics with KC. To ensure the sturdiness of our discoveries and to address potential biases stemming from pleiotropic phenomena, we executed a comprehensive set of sensitivity analyses.

Of particular note, we identified ten immune phenotypes as potential protective factors, including CD86 on monocytes, CD3 on CD28^+^ CD45RA^-^ CD8^+^ T cells, and the absolute count of CD62L on plasmacytoid dendritic cells, among others. Concurrently, 15 immune phenotypes were flagged as risk factors associated with the development of KC, encompassing FSC-A on CD14^+^ monocytes, CD27 on CD20^-^ B cells, and the percentage of CD86^+^ in plasmacytoid dendritic cells, among others. This suggests that targeted regulation of these immune cells may benefit patients with KC, however, there are few studies on such specific immune cells in the development of KC. Additionally, our study ventured into exploring the effect of 91 circulating inflammatory biomarkers on KC, revealing that a total of 4 specific inflammatory elements were associated with the disease.

Specifically, CCL28 and IL-2 demonstrated an inverse correlation with the risk of KC, suggesting a protective role against the disease. In stark contrast, FGF-23 and β-NGF emerged as predisposing elements for KC. The immune response in KC patients has received extensive attention and in-depth exploration in recent years. The immunotherapy for KC primarily involves the combined application of ICIs and targeted therapies. Traditional immunotherapy methods include IL-2 and IFN-α. While these methods are highly toxic, they were widely used in the early stages of KC treatment. Targeted targeting of protective inflammatory factors or immune cells could help improve this phenomenon.

In addition to immune cells and inflammatory factors, we also analyzed the gut microbiota, and our investigation unveiled the influence that 12 pivotal gut microbiota traits exert on KC. The association between KC and the gut microbiome is profound. Studies have illuminated substantial variations in the gut microbial composition among KC patients compared to healthy individuals. These disparities may intimately correlate with the onset, advancement, and therapeutic responsiveness of KC (18).

### The analysis of immune cell phenotypes in relation to KC

The augmentation and release of specific immune phenotypes, notably CD4 regulatory T cells, CD80-expressing granulocytes, and CD86-positive monocytes, have been pinpointed as contributors to the risk profile for KC. These biomarkers are frequently linked to an immunosuppressive environment, potentially fostering tumor expansion and advancement. For instance, the existence of CD4^+^CD25^+^Foxp3^+^ regulatory T cells has been associated with unfavorable survival rates, implying that these cells might contribute to malignancy progression by dampening anti-tumor immunological defenses (19).

Conversely, certain immune phenotypes, including FSC-A-positive CD14^+^ monocytes, CD27-positive CD20^-^ B cells, and CD86^+^-positive dendritic cells, are presumed to exert protective functions. These cells are commonly associated with augmented immune responses, potentially aiding in tumor resistance. For instance, elevated levels of dendritic cells, non-activated mast cells, and non-activated CD4^+^ memory T lymphocytes have been correlated with improved 5-year survival rates (20, 21).

The crucial function of CD4^+^ lymphocytes, particularly the regulatory subset known as Foxp3^+^ Tregs, has garnered significant focus in KC research. Studies have highlighted that an increased presence of CD4^+^CD25^+^Foxp3^-^ T cells indicate a less favorable prognosis for KC patients (19). Remarkably, Tregs exhibit the ability to transition from a CD4^+^Foxp3- state to a CD4^+^CD25^+^Foxp3^+^ phenotype, facilitated by prostaglandin E2 (PGE2), a process that may intricately contribute to the mechanisms utilized by tumors to evade the immune system’s detection (22). These findings emphasize the potential importance of Tregs in the immune evasion tactics employed by KC.

Conversely, CD8^+^ T lymphocytes occupy a central stage in orchestrating anti-tumor immune defenses. Research has demonstrated that the extent of CD8^+^ T cell infiltration serves as an independent predictor of survival rates and responsiveness to immunotherapy among KC patients (23). Additionally, distinct subsets of CD8^+^ T cells, notably those expressing TNFRSF9, display a unique blend of exhaustion and effector functionalities, leading to enhanced responses to immunotherapy.

In the realm of KC, the CD4^+^CD25^+^Foxp3- T cell subset exerts its influence predominantly through its regulatory prowess within the intricate tumor immune microenvironment. Specifically, when discussing T cells in the context of tumors, we are primarily alluding to CD4^+^CD25^+^FOXP3^+^ regulatory T cells (Tregs), which are renowned for their capacity to maintain immune tolerance by attenuating immune reactions. Tregs play a pivotal role in safeguarding tumor development by tempering the immune system’s aggressive response against the tumor, thereby promoting a favorable environment for tumor progression. This function underscores the significance of Tregs in modulating the delicate balance between immune surveillance and tumor evasion in KC (24).

CD4^+^CD25^+^FOXP3^+^ T cells possess the ability to dampen the activity of effector T cells through the secretion of inhibitory cytokines, including TGF-β and IL-10. This suppressive mechanism effectively reduces the aggressiveness of effector T cells towards tumor cells, thereby diminishing their ability to mount an effective attack against the tumor (25, 26). This mechanism helps prevent autoimmune reactions to a certain extent, but in a tumor environment, this inhibitory effect may hinder an effective anti-tumor immune response (24). Studies have demonstrated a correlation between elevated levels of Tregs and disease progression, as well as a poorer prognosis in certain types of cancer (27, 28).

While research has predominantly centered on CD4^+^CD25^+^FOXP3^+^ T cells, it is plausible to speculate that CD4^+^CD25^+^Foxp3- T cells could potentially influence tumor progression and patient outcomes through analogous mechanisms, thereby warranting further investigation into their role in the complex tumor immune microenvironment.

In the tumor microenvironment, CD8^+^ T cells frequently display impaired functionality. An illustrative example is the prevalence of melanoma antigen-specific CD8 T cells, also known as tumor-infiltrating lymphocytes (TILs), which predominantly express the PD-1 receptor, leading to a compromised functionality of these cells (29). This implies that the tumor microenvironment may hinder the activity of CD8^+^ T cells by enhancing the presence of inhibitory molecules like PD-1, fostering an environment conducive to tumor growth and progression. Additionally, tumor cells can evade the assault of CD8^+^ T cells by prompting the expression of immune checkpoint receptors, notably B7-H1 (also known as PD-L1), which serve as a means of immune evasion. Studies have shown that B7-H1^+^ melanocytes co-localize with immune cells, and this co-localization is closely associated with the presence of tumor-infiltrating lymphocytes (TILs) (30). This signifies that tumor cells possess the adaptive capability to withstand anti-tumor immune attacks by adjusting the expression levels of immune checkpoint molecules. The efficacy of CD8^+^ T cells in tumor immunotherapy stems from their precise recognition and elimination of tumor cells based on antigen specificity. As an example, IL-12 gene therapy has been shown to bolster the local proliferation of CD8^+^ T cells that are specifically targeted against tumor cells, subsequently augmenting the overall antitumor effect (31). This underscores the potential of gene therapeutic strategies to effectively stimulate and amplify the activity of targeted CD8^+^ T cell populations against tumors.

Distinct subsets within the CD8^+^ T cell population, including but not limited to Tc1 and Tc2, display diverse functional characteristics and behaviors when employed in the context of tumor immunotherapy. Tc1 cells are considered more effective in tumor immunotherapy due to their high cytotoxicity, longer survival time, and contribution to Th1-type immune regulation (32). In contrast, although Tc9 cells demonstrate lower cytotoxicity *in vitro*, they secrete different cytokines and exhibit a stronger antitumor response against advanced tumors *in vivo (* 33). This implies that diverse CD8^+^ T cell subsets contribute to tumor immunotherapy via a multitude of distinct mechanisms.

The intricate interplay of the immune system, particularly the pivotal roles played by regulatory T cells and other vital immune cell subsets, is highlighted in the development of KC. Future research necessitates a deeper exploration of the specific mechanisms underlying these immune phenotypes and how they interact to influence KC progression. Furthermore, elucidating the variations in these immune phenotypes among individuals will facilitate the development of personalized therapeutic strategies tailored to address specific risk factors.

### Analysis between circulating inflammatory proteins and KC

Interleukin-2 (IL-2) serves as a pivotal immunoregulatory molecule, eliciting a response from specific immune system components, including cytotoxic T lymphocytes and NKs, that are tasked with identifying and eliminating tumor cells within the body. Despite its widespread use in the treatment of advanced KC and melanoma, the therapeutic efficacy of IL-2 remains limited, and it may be accompanied by severe side effects (34). The administration of IL-2 has been correlated with an elevation in the concentration of soluble IL-2 receptor in patient serum, indicative of a potential enhancement in immune system function aimed at combating tumor growth (35). IL-2 is also capable of promoting inflammatory responses, particularly by enhancing the production of Th1 cells through increased secretion of IL-12. Th1 cells occupy a central position in orchestrating both anti-infective and anti-tumor immune defenses. They play a crucial role in activating and promoting the proliferation of other key immune players, including T cells and NK cells, thereby amplifying the overall immune response against pathogens and tumors. However, IL-2 therapy presents certain obstacles, primarily stemming from its capacity to activate Tregs, which in turn can dampen the effectiveness of anti-tumor immune responses (36), thereby hindering the desired therapeutic outcome. Nonetheless, through optimizing dosage and treatment protocols, it is possible to maximize the anti-tumor effects of IL-2 while minimizing its potential side effects.

Chemokines are typically responsible for attracting specific types of immune cells to sites of inflammation or infection to help clear pathogens and repair damaged tissue (37). In the case of this situation, this could mean that CCL28 can attract immune cells to the tumor microenvironment, thereby promoting immune surveillance and response to the tumor. For instance, CCL28 may contribute to inhibiting tumor growth and dissemination by bolstering the activity of T cells, as well as other integral immune cell subsets.

Although the specific roles of FGF23 and b-NGF in KC have not been directly explored, we can deduce their potential implications based on the comprehensive literature we have surveyed. FGF23, a predominantly endocrine FGF secreted by osteocytes, holds a crucial position in maintaining phosphate balance within the body, achieving this through an intricate feedback mechanism involving the kidney and vitamin D metabolism. Not only does FGF23 function in bone diseases, but it has also been found to promote tumor progression in various cancers. As an example, in the context of prostate cancer, FGF23 serves as an autocrine growth factor, fostering proliferation, invasion, and the ability for cells to grow without attachment to a substrate in laboratory settings. Conversely, eliminating FGF23 expression in living organisms leads to a decrease in tumor growth (38). FGF23 engages with αKlotho, a transmembrane protein that strengthens the attraction between FGF23 and its receptor FGFR, consequently exerting a profound influence on tumor cell signaling pathways, biological behaviors, and clinically pertinent indicators (39).

However, there is no direct information in the literature we reviewed regarding the specific role of b-NGF in KC. Typically, b-NGF is considered a factor that promotes the growth and survival of neurons, but its role in the tumor microenvironment may be related to either promoting or inhibiting tumor growth, which requires further research to clarify.

### Exploration of the relationship between gut microbiota and KC processes

This research, by conducting a thorough examination of distinctive features pertaining to the gut microbiota, has uncovered their plausible involvement in the initiation of KC processes. The gut microbiota influences the host’s health status through its metabolic activities. For instance, the production of short-chain fatty acids (SCFAs) is closely related to intestinal health, and certain biosynthetic organisms can alter the composition and activity of fecal microbiota, thereby increasing SCFA levels (40, 41). Moreover, the gut microbiota participates in diverse biosynthetic processes, including but not limited to the creation of fundamental biomolecules like amino acids and nucleotides, as well as cofactors, all vital for sustaining the host’s physiological equilibrium and functions (42).

Our findings indicate that certain metabolic pathways and bacterial species may have a protective effect on KC. Specifically, the super pathway involving Biosynthetic Pathway for L-Lysine, L-Threonine, and L-Methionine (Variant II): PWY.724, the sucrose invertase in sucrose degradation III (PWY.621), Biosynthetic Pathway I for dTDP-L-Rhamnose(DTDPRHAMSYN.PWY), and the Desulfovibrio genus and Desulfovibrio piger species in the Desulfovibrionaceae family have all shown characteristics associated with a reduced risk of KC.

These protective characteristics could potentially act as preventive mechanisms against KC development by fostering favorable metabolic activities and enhancing immune regulatory responses. For instance, the glyoxylate cycle (PWY_GLYOXYLATE.BYPASS) and the super pathway of menaquinol-6 biosynthesis I (PWY.5850) may be significant for maintaining kidney health and defending against disease invasion.

However, our findings also encompassed the identification of bacterial species that correlate with an elevated risk of KC development, including Butyrivibrio crossotus in the Lachnospiraceae family of the Firmicutes phylum, Odoribacter splanchnicus in the Porphyromonadaceae family of the Bacteroidetes phylum, Sutterella wadsworthensis in the Sutterellaceae family of the Betaproteobacteria class, and Odoribacter and Alistipes shahii in the Bacteroidales order of the Bacteroidia class. The presence of these bacteria may be related to inflammatory responses, intestinal barrier dysfunction, or other mechanisms that promote disease development.

The stearate biosynthesis II pathway (PWY.5989) has emerged as a potential risk factor for KC, implying that specific metabolic byproducts may be pivotal in triggering the disease. These findings underscore the intricate nature of the gut microbiota ecosystem and its diverse contributions to health and disease processes.

While our research sheds light on the interplay between gut microbiota and KC, there remains a need for in-depth exploration to unravel the precise biological mechanisms underlying these observed associations. Future studies should focus on how these specific bacterial species and metabolic pathways affect the host’s immune response and disease susceptibility.

Given that the gut microbiota’s composition is susceptible to modifications by dietary habits, lifestyle choices, and environmental exposures, elucidating the intricate interplay between these factors and the microbiota may pave the way for innovative approaches in the prevention and management of KC.

## Limitation

Among the limitations of our study lie the dependency on preexisting GWAS data and the imperative for subsequent investigations to decipher the biological underpinnings of the discovered associations. Future studies should focus on the direct mechanisms by which identified immune phenotypes and gut microbiota influence KC development, and how modifiable factors such as diet and lifestyle can be leveraged to mitigate disease risk.

## Conclusion

Our research underscores the critical involvement of various immune cell subsets, circulating inflammatory biomarkers, and gut microbiota signatures in KC initiation. This enriched comprehension could potentially pave the path for the development of novel strategies for KC management and therapeutic interventions.

## Data Availability

The original contributions presented in the study are included in the article/**Supplementary Material**. Further inquiries can be directed to the corresponding author.
